# The use of single-cell RNA-seq to study heterogeneity at varying levels of virus–host interactions

**DOI:** 10.1371/journal.ppat.1011898

**Published:** 2024-01-18

**Authors:** Sharmada Swaminath, Alistair B. Russell

**Affiliations:** School of Biological Sciences, University of California, San Diego, La Jolla, California, United States of America; Fred Hutchinson Cancer Center, UNITED STATES

## Abstract

The outcome of viral infection depends on the diversity of the infecting viral population and the heterogeneity of the cell population that is infected. Until almost a decade ago, the study of these dynamic processes during viral infection was challenging and limited to certain targeted measurements. Presently, with the use of single-cell sequencing technology, the complex interface defined by the interactions of cells with infecting virus can now be studied across the breadth of the transcriptome in thousands of individual cells simultaneously. In this review, we will describe the use of single-cell RNA sequencing (scRNA-seq) to study the heterogeneity of viral infections, ranging from individual virions to the immune response between infected individuals. In addition, we highlight certain key experimental limitations and methodological decisions that are critical to analyzing scRNA-seq data at each scale.

## Introduction

Viral disease is fundamentally driven by interactions of populations of viruses, often comprising a diverse set of genotypes, with populations of host cells, themselves comprised of a variety of cell types and epigenetic states. Infection outcome is dependent upon these complex, heterogeneous interactions. Until relatively recently, it was difficult, labor-intensive, and limited in terms of available experimental measurements to study infection at any level other than bulk measurements averaging effects across many different infected (and uninfected) cells. With the advent of single-cell RNA sequencing (scRNA-seq), it has now become much easier to explore complexities within viral infection.

**Fig 1:**
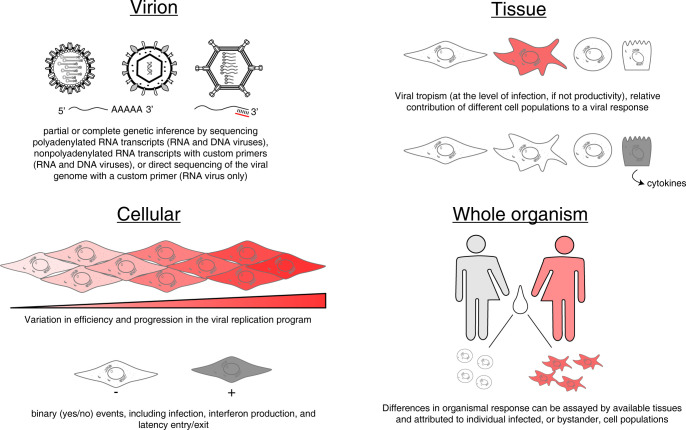
Broad examples of heterogeneity probed by scRNA-seq in the context of viral infection. Specific instances of each provided in the text of the appropriate section.

In this review, we will discuss examples of how scRNA-seq has been used to characterize heterogeneity and stochasticity in viral infection, ranging from individual virions to even understanding differences in the immune response between infected individuals (Fig 1). While a powerful new approach, as with any tool, there are important experimental limitations and methodological adjustments that are critical to understanding and interpreting scRNA-seq experiments. As we describe each level at which scRNA-seq has been applied to understanding viral infection, we also provide some considerations in terms of known limitations to the technology.

### Single-cell RNA sequencing methods

scRNA-seq has been a powerful and widely used technique to study biological heterogeneity since its development in 2009 [1]. Since then, scRNA-seq methods have advanced in sensitivity, throughput, and cost, leading to increased adoption in research and clinical settings. Existing scRNA-seq platforms are generally based on the same fundamental steps: (1) cells isolated as a single-cell suspension; (2) mRNA is captured, either in situ or from lysed cells; (3) cDNA is generated from captured mRNA and barcoded with cellular identity; (4) cDNA is prepared for sequencing; and (5) sequencing is performed (Table 1).

**Table 1:** Single-cell RNA sequencing methods discussed in this review.

Methods	Examples	Description	References
**Inidividual, sorted cells**	Smart-Seq followed by tagmentation	Individual cells isolated in microliter droplets in a standard 96- or 384-well plate, sequencing libraries generated from each independent cell. Low throughput.	Ramsköld et al., 2012 [6]
**Well-based**	Seq-Well	Cells captured in individual, subnanoliter wells with barcoded beads. Cellular barcoding of transcripts occurs during reverse transcription, material pooled for library preparation. Does not require specialized equipment beyond standard molecular biology instruments.	Gierahn et al., 2017 [16]
**Droplet-based**	InDrop, Drop-Seq, 10x Genomics Chromium	Cells co-encapsidated in droplets alongside barcoded beads in a microfluidic device. In-droplet reverse transcription provides cellular barcodes, after which droplets are combined and pooled for library preparation. Most used technology in the studies discussed in this review.	Macosko et al., 2015; Klein et al., 2015; Zhang et al. 2019 [20,21,116]
**Split-Pool**	SPLiT-Seq	Cells fixed, distributed between standard wells in a 96- or 384-well plate, and an initial barcode added to permeabilized cells via in situ reverse transcription. Subsequent ligation reactions after repooling and resplitting cells produces a combinatorial cell barcode, after which standard library preparation follows. Does not require specialized equipment beyond standard molecular biology instruments.	Cao et al., 2017; Rosenberg et al., 2018 [22,23]

scRNA-seq experiments were made possible in part due to key innovations in both cDNA synthesis and sequencing library preparation: the highly efficient Smart-Seq method, which uses template switching to amplify full-length cDNA from picogram amounts of RNA, and tagmentation using Tn5 transposase, which permits the fragmentation of much smaller amounts of material than classical shearing methods [2–10]. For the former, Smart-Seq uses 2 properties of the reverse-transcriptase derived from Moloney murine leukemia virus, the untemplated addition of cytosines at the 5′ end of mRNA transcripts, and the ability to template switch, generating cDNA that is a chimera of 2 different molecules [11]. By adding a template switching oligonucleotide, an oligonucleotide containing riboguanosine or a locked-nucleic acid form of the same at the 3′ end, this oligonucleotide can hybridize to the untemplated cytosines and be transcribed as part of the cDNA [12]. Broadly, this is a highly efficient means to add molecular handles for downstream PCR amplification of full-length cDNA. While an incredible advancement, this early effort was limited in terms of cost and throughput, as each cell was processed in an individual well of a 96- or 384-well plate through library preparation. However, we should add that, owing to the indexing of each cell performed at the tagmentation step, this method has the advantage of capturing all portions of all transcripts without the use of long-read sequencing.

One of the first efforts to parallelize and reduce the cost of this process was the use of microfluidic chips such as the Fluidigm C1 platform [13,14]. This instrument uses a microfluidic chip to capture, lyse, and perform library preparation in volumes much smaller than achievable using pipetting. However, this technology has largely been supplanted by methods that instead use droplet- or well-based platforms that accomplish even higher throughput at lower cost. A key innovation in both methodologies is the use of beads coupled to barcoded oligonucleotides, which, using a split-pool synthesis strategy, allows for every oligonucleotide on a bead to contain the same barcode, but oligonucleotides from different beads to possess different barcodes [15]. This introduces a cellular index during reverse transcription, allowing for downstream library preparation steps to be performed with cDNA from all individual cells in a single pool. By combining processing steps after initial barcoding, higher throughput, lower cost, and reduced technical noise have all been achieved relative to older technologies. However, a key limitation is that, without modification to use long-read technology, sequencing is generally limited to either 5′ or 3′ end of each transcript.

Well-based scRNA-seq methods such as Seq-Well use arrays of subnanoliter wells and a gravity-based capture of both beads and individual cells [16]. Once loaded in wells, a semipermeable membrane is applied, cells are lysed, and mRNA captured by oligo(dT) beads containing cell-specific barcodes. Beads are then recovered followed by reverse transcription of the bead-bound mRNA using the Smart-seq method and sequencing libraries with cell-specific identifiers are generated. While currently not as broadly used as droplet-based methods, discussed below, Seq-Well is relatively gentle on cells and may be more advantageous in difficult-to-handle cell types such as neutrophils [17,18]. In addition, similar to a split-pool method introduced below, this method does not require equipment beyond an array of subnanoliter wells and standard molecular biology instruments, making it easier to deploy in virological studies without the dedication of expensive equipment to a biocontainment facility.

**Fig 2:**
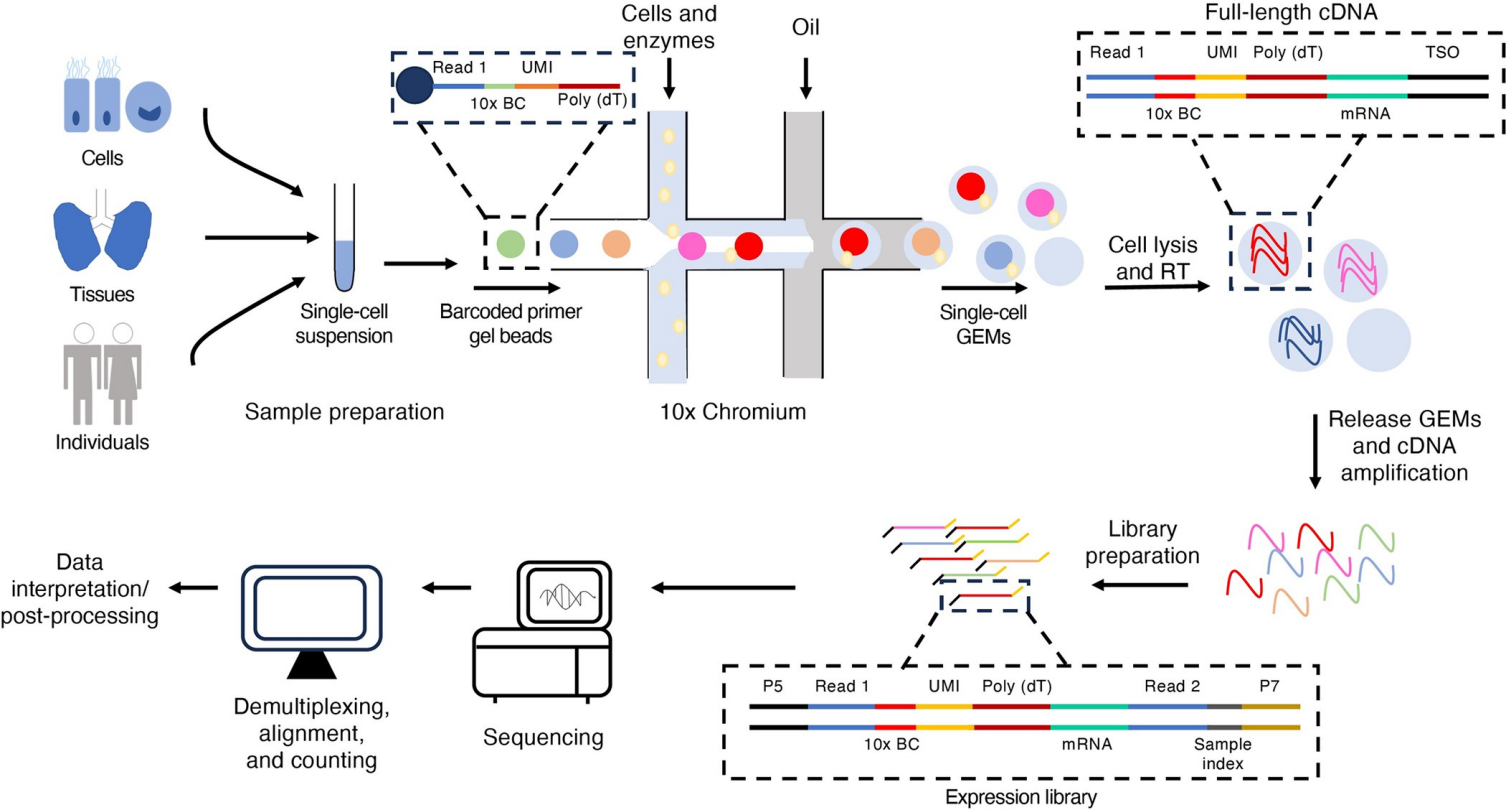
Single-cell sequencing workflow for droplet-based approaches exemplified by the 10x Genomics Chromium platform. Infected cells from cell culture, tissues/organoids, or infected individuals are dissociated into a single-cell suspension. The cell suspension is loaded onto a microfluidic chip, and cells are partitioned into nanoliter-scale Gel Beads-in-emulsion (GEMs) droplets containing barcoded gel beads and reagents for reverse transcription (RT). Following cell lysis, the beads capture the mRNA molecules. Reverse transcription (RT) by template switching using a template switching oligonucleotide (TS) generates cDNA tagged with a 10x barcode (BC) to identify the cell and a unique molecular identifier (UMI) to label the mRNA transcript. The pooled cDNA is amplified in bulk, fragmented by enzymatic fragmentation, and sequencing adapters (P5 and P7) including a sample index, are added to the fragments by PCR to generate sequencing libraries. The sequencing libraries are sequenced, and the data are analyzed by alignment and demultiplexing, following which the data are interpreted.

Drop-seq, InDrop, and the commercially available Chromium platform from 10x Genomics, collectively, are droplet-based methods of scRNA-seq (Fig 2) [19–21]. Microfluidic chips are used to generate picoliter droplets containing cells (loaded probabilistically and assumed to follow a Poisson function of occupancy) and barcoded beads. These droplets contain cell lysis and reverse transcription reagents. While maintaining droplet integrity, cells are lysed and reverse transcribed to generate cDNA in a standard thermocycler. As the cDNAs are already tagged with a cell-specific barcode, emulsions are broken and sequencing libraries are prepared by standard methods. Cell-specific barcodes added during reverse transcription are from oligonucleotides coupled to beads, either as an oligo(dT) and marking the 3′ end of transcripts, or in the template-switching oligonucleotide, labeling the 5′ end of transcripts.

Using a similar split-pool philosophy as barcoded bead generation to identify individual cells, but using the cell itself as a reaction vessel, is split-pool sequencing, such as SPLiT-seq or SCI RNA-seq [22–24]. Rather than lysing the cell, cells are first fixed and permeabilized. Then, cells are distributed between individual wells of a 96-well plate, with multiple cells per well. A barcode can be added at this point by reverse transcription, with each cell within a well sharing a barcode, but distinct barcodes between wells. Following within-cell reverse transcription, cells are pulled from wells, mixed, and redistributed again between wells. An additional well-specific barcode is added at this point by ligation. By performing multiple additional rounds of barcoding after “reshuffling” the cells, each cell is tagged with a unique combination of barcodes, and, thus, cDNA are reassigned to an individual cell after sequencing. After barcoding, cells are lysed, and standard library preparation followed. Split-pool sequencing has the potential to be highly scalable, with theoretical barcode diversity increasing exponentially with the addition of each barcoding step. Additionally, the use of fixed cells and, as with well-based approaches, the lack of specialized equipment, makes this approach potentially more appealing when working with infectious agents.

While we generally make no distinctions in the remainder of the review on which technology was used to generate observations in terms of viral infection and heterogeneity, we do note that by far the most frequent technology used has been droplet-based sequencing (described in Fig 2). We therefore largely restrict our discussions of experimental considerations and caveats to that technology.

### Virion-level heterogeneity

It has been appreciated for quite some time that viral populations are heterogeneous [25]. Some of the earliest observations in virology were that the number of physical particles in viral preparations greatly exceeds that of infection-competent particles alone. While some of these particles are merely empty capsids, not relevant to the technologies we describe here, many bear mutations or other genetic defects that render them incapable of completing the viral life cycle. Curiously, despite outnumbering their replication-competent counterparts, the consensus sequence across a viral population is generally fit (safe for populations enriched for defective viral genomes, discussed briefly below), indicating a myriad of different genetic defects underlie the failure to replicate rather than a single stereotyped defect that rises to consensus.

These unfit particles have represented a sort of “dark matter” in experiments [26–29]. They may still enter cells and can influence features such as the induction of an innate immune response but are difficult to assess using bulk methodologies that largely focus on replication-competent components, particularly those that rise to consensus, of a viral population. As viral genomes are highly compact, or themselves can serve as the messages for protein production, sequencing of mRNA pools in a cell can provide insight into the totality of viral genotypes, replication competent and otherwise. This assumes the defect itself does not prevent transcription—or that the genome itself is an RNA molecule—however, this limitation still provides access to biological features that were generally difficult to access by older methodologies.

The capacity to explore individual virion genetic variation using modern scRNA-seq, replication-competent or otherwise, has been perhaps most extensively (but not exclusively) realized in the study of influenza A virus [30–36]. As this virus possess a relatively small genome of approximately 13.5 kb, split across 8 RNA molecules, the longest of which is still relatively short at 2.3 kb in length, and nearly all of which is transcribed into capped, polyadenylated mRNA, very few modifications are required to use preexisting single-cell approaches to access viral genetic heterogeneity in a standard scRNA-seq workflow [37]. Indeed, nonsegmented RNA viruses would present a considerable challenge for genetic inference as it is difficult to achieve full-length cDNA synthesis from long, structured RNA molecules, and DNA viruses would present their own challenge as regions that are not transcribed cannot be measured by these approaches. While only 2 manuscripts thus far have sequenced the entire 5′ to 3′ ends of viral transcript in infected cells, several have used partial genetic information to inform studies of viral variation, including features such as reassortment [38].

The most commonly used workflow in scRNA-seq is to sequence either the 5′ or 3′ ends of transcripts. This provides the ability to recover both the cell-identifying barcode (in either the template-switching oligonucleotide or the oligo(dT) primer) as well as sufficient sequence information to assign transcripts. The key limitation requiring fragmentation and end-choice is due to both the read-length limitation of Illumina sequencing, as well as length requirements for efficient bridge amplification in an Illumina flow cell [39]. From this partial genetic information, several studies nevertheless were able to identify architectural changes to viral genomes, for instance, large internal deletions in influenza A virus [31,33,34]. These deletions form the basis of defective viral genomes (DVGs, also called nonstandard viral genomes or nsVGs), which are associated with a general loss of viral fitness as well as the induction of an innate immune response [40,41]. Critically, these studies not only identified these species but also were able to assay them with respect to a heterogeneous host response, indicating that while they are associated with induction of an innate immune response, they are neither necessary nor sufficient to induce interferon but instead increase the probability of its expression nondeterministically.

### Experimental considerations for the study of virions; read-length limitations and alternate priming strategies

Several considerations should be taken into account if using single-cell transcriptomics to assay viral genetic heterogeneity alongside other features of interest. The first is the limitation of the standard workflow to 5′ or 3′ end sequencing. In addition to the obvious—that variant capture will largely be limited to sequences in these regions—the “width” of the coverage window is largely dependent on sequencing depth. For viral reads, this will, in turn, depend on the level of viral transcripts in an infected cell. Thus, successful genotyping, including architectural variation, is impacted by the level of viral transcripts [34]. This can lead to certain incorrect conclusions; for instance, if the probability that a large deletion in the viral genome is detected is proportional to the level of coverage, then that deletion will be found to be correlated with high levels of viral transcription regardless of its actual biological impact.

Solving the issue of partial coverage, during the preparation of sequencing libraries, full-length, tagged cDNA is generated prior to fragmentation. This material can be sequenced by long-read technologies, such as PacBio SMRT or nanopore sequencing. This methodology has been termed “ISOseq” (ScISOr-Seq in single cells) and has been used to annotate isoform differences in both bulk and single-cell sequencing [42,43]. A newer method, named HIT-ScISOr-Seq, further uses concatemerization to increase the throughput of long-read sequencing to more comprehensively measure full-length mRNA across an entire scRNA-seq experiment [44,45]. Use of a targeted ScISOr-Seq workflow to call viral genotypes has been used in 2 different manuscripts for influenza A virus, and similar approaches could be extended to other viral populations [35,36]. One key artifact, though, that should be considered is that some genotypes will be erroneously assigned to certain cells owing to template switching during reverse transcription or chimera formation during PCR [46]. One possible solution is to use populations with known variants that differ at the 5′ and 3′ end of transcripts, thus allowing for an empirical measurement of these artifacts [35].

Additionally, not all viruses generate capped, polyadenylated messages. This is particularly true of RNA viruses outside of the Orthomyxoviruses. As current scRNA-seq protocols almost exclusively use oligo(dT) priming, these messages would be missed. One potential solution is to use a splinted oligonucleotide, appending a unique sequence to capture virus-specific transcripts. This was used by Saikia and colleagues to sequence Reovirus genomes, a dsRNA virus that does not generate polyadenylated transcripts [47]. Regarding cap structures, while a wealth of literature suggests that the template-switching activity is limited to canonical 7-methylguanosine cap structures, more mechanistic studies have demonstrated that while this behavior is more efficient on 5′ methylated caps, it can still work on alternative 5′ ends, extending full-length cDNA synthesis and amplification out to alternate RNA structures that may be present in viral transcripts or genomes [48].

Lastly, the standard workflow used for most scRNA-seq approaches uses readmappers that, while appropriate for mapping spliced host transcripts with little variation between reference and reads, are inappropriate for mapping viral diversity. For instance, the most commonly used readmapper for scRNA-seq data, STAR, attempts to map disjoint (gapped) reads using a combination of preassigned splice junctions or scoring models that take into account general features of splice junctions [49]. When applied to architectural variation in viral populations, such as recombination or large deletions, these read mappers will inappropriately apply these scoring models. It is therefore critical to use specific, purpose-built pipelines, such as ViReMa or VODKA2, for mapping viral variation, and potentially regenerating viral consensus sequences when diversity significantly diverges from the chosen reference sequence [50,51].

### Intercellular variation

Some of the first uses of scRNA-seq in virology were to assay heterogeneity between cells in tissue culture infections of either immortalized or patient-derived primary cells [30,32,35,52–54]. Despite the relative uniformity of these cells, there exists considerable variation in even one of the simplest models of infection. Examples in terms of viral measurements include the amount of viral transcript made, entry and exit into latency, and progression through complex viral life cycles. On the host response to viral infection, measurements can be made between bystander and infected cells, correlations of host response and viral abundance, and, lastly, engagement of the host innate immune response.

With respect to viral measurements, one of the most stark observations across multiple viral families is the extreme variation in the fraction of virally derived reads within a cell. Cells often vary in terms of viral transcript abundance by as much as several orders of magnitude, as observed in flavivirus, influenza, and coronavirus infections, to name a few [32,52,54]. Similar heterogeneity has also been observed in viral burst size, from early measurements in bacteriophage out to more recent measurements in influenza, although the relationship between transcript variance and burst size is nonlinear, in part due to the fact that many infections are nonproductive despite completing a large portion of the viral replication program [36,55,56]. So, while population-level bulk measurements can establish per cell productivity, as well as the average fraction of transcripts that are derived from viruses, a “boots-on-the-ground” view demonstrates these averages miss the high degree of heterogeneity in actual infections. This has implications in appropriate modeling of viral replication and, if burst size is variable even within a given genetic variant, has implications for effective viral population size and the evolutionary rates within individual infections.

Using this heterogeneity as a means to understand the host response, the massive breadth in viral transcription can then be correlated with host gene expression. After all, one would hardly expect a cell wherein >50% of the transcripts are derived from a viral infection to be equivalent to one where only one out of every thousand transcripts is viral. The most straightforward analysis is to look at genes that vary in expression in a manner correlated with viral expression. This type of analysis has been broadly useful for at least 2 reasons: that these genes themselves may be causal with respect to either pro- or antiviral strategies, and, independent of direct impacts on the virus, that they represent stressors on host cells imposed by viral infection. An example of the former, high expression of interferon-stimulated antiviral genes correlates with lower viral transcripts for HSV-1 and West Nile virus infections, indicating successful engagement of host defenses as well as demonstrating that the breadth of viral outcomes are driven, at least in part, by host restriction mechanisms [53,57,58]. For the latter, the massive metabolic burden placed on cells by viral infection can leave a distinct signature that is readily revealed by correlating such pressures with viral burden even within a single experiment. For instance, oxidative and ER stress pathways have been observed to correlate with viral infection across multiple phylogenetically distinct viral families, perhaps due to the relatively high energetic and protein-folding demands placed on a cell by the massive increases of nucleic acid and protein biosynthesis required for viral replication [32,52,57,58].

Beyond these straightforward correlations, scRNA-seq has also allowed the study of highly stochastic, bimodal (yes/no) events in viral infection. On the viral side, a fantastic example of this is the study of latency. The earliest example of which we are aware of this use was in the study of HCMV infection in primary cells, wherein more precise correlations of latency, host gene expression, and viral programs were able to be established by the capacity to separate out subpopulations of cells to ensure that correlated features coexisted within the same infected cell—critical as while such features may correlate at the population level, that does not necessarily mean that such programs are occurring within the same cell [59].

On the host side, one of the best examples of a bimodal event is the engagement of an antiviral response. The production of interferons in response to viral infection has been known to be highly stochastic for quite some time, but the features that lead to this stochasticity have been very difficult to study [60–62]. Using scRNA-seq, individual cells producing interferon can be identified [63,64]. This, in turn, can be correlated with viral infection, levels of viral transcripts, and, as discussed in the prior section, viral genetic variation to determine what features may lead to the induction of a response. Thus far, it has been illuminating that while there is a considerable contribution of viral variation to the induction of an innate immune response, even stimulatory variants within a population tend to induce a stochastic, if more common, production of interferons, indicating a role for chance events or preexisting host cell heterogeneity [34,65].

Lastly, in addition to profiling standing variation within a single tissue culture infection, single-cell transcriptomics permits the simultaneous profiling of many CRISPR modulations within a single experiment, allowing one to deliberately introduce variation and perform a highly parallel genetic analysis. One method that accomplishes this is Perturb-seq [66]. Perturb-seq has been used to identify host factors that influence viral replication and transcription, determining how particular perturbations may enhance, or repress, the viral replication program, as has been performed for Severe Acute Respiratory Syndrome Coronavirus 2 (SARS-CoV2) [67]. Moreover, while likely restricted to only DNA viruses, Perturb-seq has also been used to directly profile CRISPR perturbations to a herpesvirus genome, human cytomegalovirus, reducing an otherwise laborious process of individually mutating and assessing the effect of each individual viral gene to a single one-pot experiment [68].

### Experimental considerations for the study of intercellular variation; lysis and batch effects

A number of modifications to the standard single-cell workflow are even useful in interpreting these relatively simple models of infection. One problem that particularly plagues an understanding of viral infection models by scRNA-seq is the contribution of extracellular/misassigned RNA (also referred to as ambient RNA). As indicated in the prior section, there is considerable interest in bimodal events in viral infection, which can even include the probability that a cell itself is detectably infected. Droplet sequencing methodologies have significant amounts of reads contributed from the “soup,” a mixture of misassigned reads due to template switching and PCR chimeras, as well as extracellular RNA from cell lysis, membrane vesicles, and potentially even viral particles themselves. This may particularly pose a problem for cytopathic viruses, which may be overrepresented in the “soup.” This creates a challenge when identifying whether cellular responses are directly due to viral infection or due to signaling from infected cells in terms of the “bystander” population, as uninfected cells may nevertheless be observed to be associated with viral RNA.

While true negatives can never really be identified in scRNA-seq data, it is critical to set some kind of threshold for true positives. In other words, where is the limit of detection? This limit cannot be extrapolated across even individual sequencing runs from the same laboratory, as the degree of contamination is highly idiosyncratic and sample dependent. There exist some methods to interpolate these contamination levels in datasets, such as SoupX, although caution should be used as these methods rely on distinct transcriptional clusters of cells that may not reliably exist in tissue culture models of infection [69–72]. Another method is to actually measure the contamination rate, which can be achieved by adding in a cell type that possesses none of the transcripts of interest and measuring their rate of presence (for instance, by adding uninfected canine cells to infected human cells immediately prior to processing) [35].

Secondly, batch effects can also play a role when comparing across experimental treatments [73]. Regression analyses often find that replicate number is a significant source of signal in single cell analyses [74]. This is a trickier problem to overcome, and there are likely multiple nonoverlapping reasons for batch-to-batch variation, which may include, but is unlikely to be limited to, ambient RNA contamination. Inclusion of multiple replicates of different treatment conditions, and use of a regression analysis to identify genes that tend to vary based on replicate rather than experimental treatment, can partially address these issues with scRNA-seq [75–78]. Alternatively, as batch effects tend to be limited to a particular scRNA-seq run, cells under different experimental conditions can be labeled using “hashing” reagents, which are either an antibody coupled to a short oligonucleotide or a compound coupled to a short oligonucleotide that can embed in cell membranes, permitting multiplexing of experimental conditions in a single experimental run by marking cells from each condition in a way that is recoverable during sequencing [79].

### Tissue-level heterogeneity

While simple, homogenous tissue culture models of infection can be informative, real infections are significantly more complex. The fact that infections occur in the presence of multiple divergent cell populations, which themselves communicate extensively with one another is missing. This type of complexity lends itself well to scRNA-seq, as prior studies of tissue-level phenomena were either limited by averaging over potentially wildly divergent individual cellular responses or, when cell-level heterogeneity could be resolved by microscopy or flow cytometry, limited to a relatively small number of targeted observations.

Unlike in homogenous tissue culture experiments, we would anticipate tissue infections to have significant variability in terms of not only what cells are present but also what cells are infected. This, for instance, can provide insight into how infections are successfully controlled by cell intrinsic innate immunity. For instance, when comparing infected and bystander cells during influenza and Ebola infections in in vivo animal models, bystander cells exhibit significantly higher expression of interferon-stimulated genes [33,80,81]. This could represent both viral suppression in infected cells, as well as an inability of these viruses to spread within tissues engaged in active interferon signaling. Similarly, changes in cell infectivity can also be assessed by scRNA-seq during antiviral drug treatment [82].

Comparing infected, and bystander, cells in tissues also can provide insights into viral tropism. While scRNA-seq measurements do not meet the classical definition of tropism, which is the productive infection of a cell type, they provide identification of cells that support successful viral entry and engagement of some portion of the viral transcriptional program. This can also be seen as an advantage, as both productively, and nonproductively, infected cells can contribute to overall pathology and disease progression. This method was used to support tropism of SARS-CoV2 in both human and animal tissues, including ex vivo human lung models, not only confirming angiotensin converting enzyme 2 (ACE2) as the predominant receptor, and identifying cell-specific expression of this receptor, but also demonstrating ACE2-independent entry into certain cell populations [83–85]. Not limited to SARS-CoV2, cells susceptible to infection have been studied using scRNA-seq for astrovirus, rubella, and influenza, among others [80,86,87].

### Experimental considerations for the study of tissue-level variation; isolation effects and biases

While generation of single-cell suspensions required for scRNA-seq is often straightforward in homogenous tissue culture infections, there is a considerable challenge to generating them from complex animal or human tissues. For instance, neurons are incredibly fragile and possess a large number of intercellular contacts and are incredibly difficult to isolate without their destruction. One solution for such tissues is to perform single-nuclei RNA-seq (snRNA-seq), rather than scRNA-seq [88–91]. Nuclei are morphologically simple, making their isolation for RNA-seq easier than intact cells. snRNA-seq compares well with scRNA-seq data, although reads should be mapped to intronic as well as exonic regions owing to the increased presence of unprocessed mRNA in snRNA-seq [92,93]. A caveat to this approach somewhat unique to virology is that it would be limited to viruses that replicate in the nucleus, as cytoplasmic RNA would fail to be captured.

In addition to the challenges of generating a single-cell suspension from a complex tissue, choice of dissociation protocol can influence experimental results [94,95]. There is no perfect way to capture all cells, and protocols may fail to solubilize all populations equivalently, or else, if using harsh detergents to address tough extracellular matrix, may result in the loss of more sensitive cells. There is no universal solution to this issue, but analysis of scRNA-seq data should be performed with an awareness that cell distributions may not reflect their true biological abundance but only their representation in the processed sample.

With regard to identifying different cell populations, cell type annotation can be accomplished using known markers or unsupervised clustering algorithms [96–100]. However, these processes possess their own biases. Cells with unusually low or high gene content, amplification biases, and ambient RNA contamination, all impacting downstream clustering [101]. Prefiltering, that is removing cells with unusually low or high gene counts, or unusually large amounts of mitochondrial transcripts or a high degree of intron retention, can address some of these issues but may also remove true biological signal unique to viral infections such as general host transcriptome suppression. Another means of addressing these difficulties is to use multimodal datasets with included cell surface marker data, using oligonucleotide labeled antibodies that are resolvable in scRNA-seq in order to combine targeted, hypothesis-driven measurements with massive scRNA-seq datasets [102]. Rare cell types, which themselves can have a massive impact on disease progression, are also difficult to identify using standard clustering algorithms. Preenrichment of rare cells using fluorescence activated cell sorting can also help to address this issue but requires preexisting markers for populations of interest.

### Interindividual variation

Disease outcome can vary widely between individuals. While this variation in outcome is broadly accessible to bulk methods, a key component of this variation is differences in individual cellular response as well as relative proportions of different immune cells during infection. scRNA-seq provides sufficient resolution to unravel these differences, providing deeper knowledge into disease severity and progression.

A key component of interindividual variation is the immune response to infection. For instance, in HIV infection, a minority of HIV^+^ individuals, termed “elite controllers,” largely control viral growth. scRNA-seq of these individuals was able to identify elite controller–specific signatures in HIV-specific CD8^+^ T cells in the lymph node, which indicated a role for noncytolytic functions in control of this virus [103]. An additional example, ex vivo infection of peripheral blood mononuclear cells (PBMCs) of individuals with divergent genetic ancestry was able to find significant differences between a population of European ancestry and a population of African ancestry in terms of the interferon response when challenged with influenza virus [104]. With an eye towards a potential clinical application, scRNA-seq has also been applied to biomarker study, with the identification of monocyte and B cell–specific signatures associated with progression to severe dengue [105].

Similarly, scRNA-seq has also been used to understand progression of Coronavirus Disease 2019 (COVID-19) disease. scRNA-seq on PBMCs from COVID-19 patients have demonstrated lymphopenia, T-cell exhaustion, expanded myeloid compartments, and an association of expanded classical monocyte population with severe disease [17,106]. In addition, scRNA-seq analysis of bronchiolar lavage fluid samples from COVID-19 patients found a shift in cell populations consistent with increased inflammation in those individuals whose course of disease was more severe [107]. Moving from immune responses, scRNA-seq has also been used to explore the response to antiviral therapy, such as in HIV^+^ patients [108].

### Experimental considerations for interindividual variation; batch effects and tissue accessibility

An issue common to both bulk methodologies and scRNA-seq when exploring clinical disease is tissue accessibility. Many tissues cannot be accessed ethically premortem, limiting our ability to study disease. Work on peripheral cells, such as PBMCs, can give insight into systemic disease as well as immune state (and provide biomarker information), but we remain limited in terms of studying disease at the site of infection for many different viruses. Organoids, or organ-on-a-chip approaches, are attempting to address this current shortcoming [109].

Batch effects, discussed in a prior section, are also a significant concern when comparing across individual responses. However, interindividual samples have an additional means of sample hashing unavailable in tissue-culture experiments. As scRNA-seq captures genetic information on not only the virus but also the host, this genotype-level variation can be used to identify which cells derived from which individual. This markerless batching of cells from multiple individuals into a single RNA-seq dataset can help to aid in variation in processing and sequencing [110].

## Conclusions

There has been an increased adoption of scRNA-seq in virology, as well as broadly in the field of molecular cell biology and even in clinical science. Continued development of these methods and decreases in sequencing cost will likely continue, making this yet another tool for use in molecular virology. Despite the limitations in assessing the heterogeneity at the various levels during viral infection, a number of experimental adjustments and specific pipelines have been devised to carefully evaluate viral and cellular heterogeneity (Tables 2 and 3). With the use of carefully considered experimental pipelines, scRNA-seq can serve as both a powerful hypothesis generation tool, as well as a means of uncoupling whether features correlated at the population level co-occur in individual infected cells. Use of scRNA-seq on clinical samples has also provided significant insight into the nature of viral disease, expanding from transcriptional and cytokine signals out to how individual populations of circulating immune cells are reshaped during and after viral infection.

**Table 2:** Table describing the caveats and experimental modifications that can aid in scRNA-seq to assess heterogeneity at different levels.

Experimental questions	Limitations	Controls
**Virion-level heterogeneity**	Partial coverage	Generation of full-length transcripts by template switching and sequencing full-length by scISO-seq and HIT-scISO-seq
	Lack of polyadenylated viral transcripts to capture	Use of splinted oligos with virus-specific sequences
	Using read mappers like STAR is inappropriate for mapping viral diversity	Use of more purpose-built pipelines like ViReMa and VODKA2 for mapping viral variation
**Cellular-level heterogeneity**	Misassigned reads due to template switching, PCR chimeras, and extracellular RNA from cell lysis	Using control cell lines that do not have transcripts of interest
		Methods like SoupX to remove ambient RNA
	Batch variations between experiments (impacts all experiments but addressed in this section of this review)	Regression analysis of genes varying between replicates
		Cell Hashing with barcoded antibodies enabling multiplexing of experimental conditions
**Tissue-level heterogeneity**	Isolation of intact single cells from complex tissues	Single-nuclear RNA sequencing for complex tissues
	Cell clustering—affected by the quality of isolated cells, amplification biases, and misassigned reads	Prefiltering the data
		Use of oligonucleotide-labelled antibody to generate multimodal dataset
	Rare cell groups	Preenrichment of rare cell types by using fluorescent-associated cell sorting
**Individual-level heterogeneity**	Accessibility of tissues from infected individuals	Humanized organ-on-a-chip models from relevant individual cells
	Batch variation between experiments	Use genetic variation between individuals to assign cells to the individual

**Table 3:** Example computational tools useful in the analysis scRNA-seq datasets during the study of virus–host interactions.

Software	Purpose/Description	References
**STAR**	Read mapping software with support to specifically map spliced reads.	Dobin et al., 2013 [49]
**ViReMa, VODKA**	Used to map nonstandard viral genomes, such as DVGs and recombination within viral species	Routh et al., 2014; Achouri et al., 2023 [50,51]
**CellRanger**	Commercial software from 10x genomics for parsing single-cell sequencing.	10xgenomics.com
**SoupX, CellBender**	Software used for elimination of technical artifacts in scRNA-seq such as ambient RNA and chimeric PCR artifacts.	Young et al., 2020; Fleming et al., 2023 [71,117]
**Viral-Track**	It relies on the STAR aligner to map the reads of scRNA-seq data to both the host reference genome and an extensive list of high-quality viral genomes. Further, specific annotation of infected versus bystander cells enables the identification of DEGs between infected and bystander cells.	Bost et al., 2020 [118]
**Seurat, Monocle**	Used to analyze scRNA-seq data, including dimensionality reduction, batch correction, and differential gene expression. It also uses unsupervised clustering algorithms to assess cell states and is useful in detecting novel cell states.	Hao et al., 2023; Cao et al., 2019 [119,120]

DEG, differentially expressed gene; DVG, defective viral genome; scRNA-seq, single-cell RNA sequencing.

Looking to the future, barriers to performing scRNA-seq are increasingly becoming lower, with methods being developed that do not even require instrumentation or microfluidics to perform cell encapsidation [111]. Besides these engineering and chemical advances, there is increasing development of bioinformatic pipelines and tools that are appropriate for understanding viral infection. In addition to scRNA-seq, there is increasing use of spatial transcriptomics, with technologies beginning to approach single-cell resolution, permitting the same explorations described here but with an additional aspect of understanding where, in complex tissues, events are occurring [112–115]. We hope that the examples, and considerations, we have provided here aid the field in proceeding to use both scRNA-seq as well as newer technologies to continue to explore fundamental questions in the field of virology.
